# Virulence Factors and Molecular Identification of *Candida* Species Causing Candidemia in Honduras

**DOI:** 10.3390/jof11070470

**Published:** 2025-06-20

**Authors:** José Fernando Chávez, Bryan Ortiz, Roque López, Carlos Muñoz, Kateryn Aguilar, Isis Laínez-Arteaga, Celeste Galindo, Luis Rivera, Manuel G. Ballesteros-Monrreal, Kathy Montes, Mauricio Hernández, Asly Villeda Barahona, Stephanie Hereira-Pacheco, Gustavo Fontecha

**Affiliations:** 1 Instituto de Investigaciones en Microbiología, Facultad de Ciencias, Universidad Nacional Autónoma de Honduras, Tegucigalpa 11101, Honduras; jchavezv@unah.hn (J.F.C.); kateryn.aguilar@unah.edu.hn (K.A.); lrivera@unah.edu.hn (L.R.); delmer.hernandez@unah.edu.hn (M.H.); asly.villeda@unah.hn (A.V.B.); 2 Laboratorio Nacional de Vigilancia, Secretaría de Salud de Honduras, Tegucigalpa 11101, Honduras; rohlome@gmail.com (R.L.); carlosmicro1728@hotmail.com (C.M.); 3 Laboratorio de Bacteriología, Hospital Mario Catarino Rivas, San Pedro Sula 21101, Honduras; izla04@gmail.com; 4 Departamento de Microbiología, Instituto Hondureño de Seguridad Social, Tegucigalpa 11101, Honduras; celestegalindom@gmail.com; 5 Departamento de Ciencias Químico-Biológicas y Agropecuarias, Universidad de Sonora, Campus Caborca, Hermosillo 83000, Mexico; manuel.ballesteros@unison.mx; 6 Departamento de Microbiología, Instituto Hondureño de Seguridad Social, San Pedro Sula 21101, Honduras; kathy.montes@unah.hn; 7 Departamento de Biología Celular y Genética, Escuela de Biología, Facultad de Ciencias, Universidad Nacional Autónoma de Honduras, Tegucigalpa 11101, Honduras; 8 Laboratorio de Interacciones Bióticas, Centro de Investigación en Ciencias Biológicas, Universidad Autónoma de Tlaxcala, San Felipe Ixtacuixtla, Tlaxcala 90120, Mexico; shereirap@gmail.com

**Keywords:** candidemia, Honduras, *Candida* spp., virulence

## Abstract

Invasive fungal infections (IFIs), primarily caused by *Candida* species, represent a significant global public health concern due to their high mortality rates and growing antifungal resistance. In Honduras, data on their epidemiology remains scarce. This study aimed to characterize *Candida* species associated with candidemia and assess key virulence factors. A total of 80 clinical isolates were collected from four hospitals in Honduras’s major cities, Tegucigalpa and San Pedro Sula. Identification was performed using both phenotypic and molecular methods. Hemolytic activity, phospholipase and protease production, and biofilm formation were evaluated. *C. albicans* and *C. tropicalis* were the most prevalent species (30% each), followed by *C. parapsilosis* (27.5%). Phenotypic methods misidentified 13.8% of the isolates. Most strains (96.3%) exhibited strong hemolytic activity. *C. albicans* showed the highest phospholipase activity, while *C. tropicalis* was the most robust film producer. These findings highlight an evolving epidemiological landscape characterized by an increasing prevalence of non-*albicans Candida* species, often less susceptible to antifungal agents, and diverse virulence profiles such as strong biofilm formation. This underscores the clinical need for accurate species-level identification through molecular diagnostics and ongoing surveillance to guide targeted antifungal therapy and enable early, locally adapted interventions.

## 1. Introduction

Invasive fungal infections (IFIs) represent a serious global health threat, causing an estimated 3.8 million deaths annually [1]. With mortality rates reaching up to 68%, IFIs surpass the global mortality burden of diseases such as malaria and tuberculosis [1,2]. According to the criteria established by the European Organisation for Research and Treatment of Cancer/Mycoses Study Group (EORTC/MSG), an IFI is defined as the presence of filamentous fungi and/or yeasts in a normally sterile body site [3,4]. Among these, candidemia—the presence of *Candida* species in the bloodstream [5]—is the most common form of invasive mycosis worldwide. It is associated with an estimated 1.5 million cases and up to one million deaths annually, with reported mortality rates as high as 63.6% [1].

*Candida* species are the leading cause of fungal infections in humans [2], with *C. albicans* being the most prevalent, accounting for approximately 50% of candidemia cases [6]. However, the epidemiology of *Candida* infections has become increasingly diverse, with a notable rise in cases caused by non-albicans species such as *C. glabrata*, *C. tropicalis*, *C. parapsilosis,* and *C. krusei* [6,7,8]. More recently, *C. auris* has emerged as a significant cause of candidemia and, in some settings, has become the predominant species [9]. The increasing incidence of non-albicans *Candida* (NAC) species has been attributed to multiple factors, including the selective pressure exerted by prolonged antifungal use, particularly fluconazole, the widespread use of invasive medical devices, and the growing number of immunocompromised patients [10,11]. These species often show reduced susceptibility to commonly used antifungals and express variable virulence traits, posing additional clinical challenges [12,13].

The mortality associated with *Candida* species in cases of candidemia is estimated to be approximately 40.4%, although this rate varies depending on the species involved. For instance, *C. tropicalis* exhibits the highest species-specific mortality, reaching up to 63.6%, while *C. auris* has reported mortality rates ranging from 39% to 45% [14,15]. This high mortality is linked to multiple factors, including a growing population of susceptible individuals with predisposing conditions. These include the use of immunosuppressive therapies, broad-spectrum antibiotics, hormonal treatments, and the presence of invasive medical devices. Such devices, like central venous catheters and urinary catheters, serve as direct entry points for *Candida* and facilitate biofilm formation, which hinders antifungal treatment and promotes persistent infection. Moreover, infections such as HIV and SARS-CoV-2 weaken host immunity, further increasing the risk of invasive fungal infections and contributing to poor clinical outcomes [5,16,17].

The ability of *Candida* species to infect various host tissues and colonize different surfaces is driven by a diverse array of virulence factors, including adherence, dimorphism, thigmotropism, phenotypic switching, biofilm formation, and the production of hydrolytic enzymes such as hemolysins, proteases, and phospholipases, all of which contribute to tissue invasion and damage [18,19]. Given the intrinsic differences in antifungal susceptibility and pathogenic potential among *Candida* species, accurate identification and characterization of virulence factors are essential in clinical settings. These actions not only enable more effective therapeutic management through species-directed antifungal treatments but also strengthen epidemiological surveillance and the understanding of species distribution and clinically relevant virulence patterns [6,20,21]. This study aimed to assess the current distribution of *Candida* species associated with invasive fungal infections in Honduras and to characterize their virulence profiles through the evaluation of hydrolytic enzyme activity and biofilm formation. Epidemiological data on *Candida* species involved in IFIs remain scarce in Honduras. Given the country’s substantial burden of fungal diseases, this study is critical to improving both the prevention and management of these infections. To our knowledge, this is the first investigation of its kind in Honduras, conducted as part of an epidemiological surveillance program involving multiple healthcare centers across the country.

## 2. Materials and Methods

### 2.1. Candida Isolation Procedures

A surveillance program was established in collaboration with the National Surveillance Laboratory (LNV) of the Honduran Ministry of Health (SESAL) to identify and characterize *Candida* species isolated from cases of IFIs reported across the country. As part of this initiative, tertiary care laboratories within the national health system were invited to participate by sending *Candida* isolates from sterile anatomical sites to the LNV-SESAL. Four laboratories joined this initiative. The isolates submitted by these centers underwent confirmatory identification and virulence profiling to strengthen epidemiological surveillance and support decision-making in the management of these infections.

### 2.2. DNA Extraction and Molecular Identification of Candida Complexes

**Figure 1:**
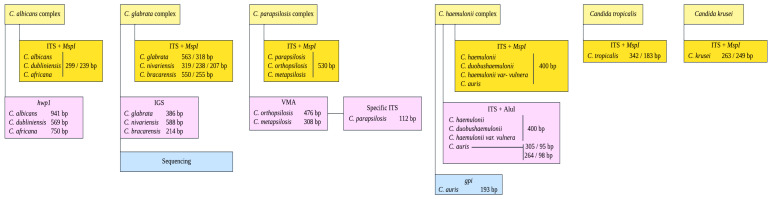
Flow diagram depicting the molecular methods used to identify species within four *Candida* complexes (i.e., *C. albicans*, *C. glabrata*, *C. parapsilosis*, and *C. haemulonii*).

All isolates were cultured individually in YPD liquid medium (1% yeast extract, 2% peptone, and 2% dextrose) and incubated at 30 °C for 24 h with continuous shaking at 200 rpm. Then, DNA extraction was performed using a previously established protocol [22,23], which involves mechanical disruption of yeast cells with a Disruptor Genie System (Scientific Industries, Inc., Bohemia, NY, USA) and 0.1 mm glass beads (BioSpec Products, Inc., Bartlesville, OK, USA), followed by DNA recovery through precipitation with organic solvents. A PCR-RFLP method was used to identify yeast isolates (i.e., *Candida* complexes) by amplifying the internal transcribed sequence (ITS) of the ribosomal region and performing DNA digestion using the enzyme MspI [22]. To differentiate between cryptic species, we followed the algorithm described in Figure 1. Yeasts identified within the *C. albicans* complex by PCR-RFLP were classified according to the protocol established by Romeo et al. (2008), which is based on size polymorphism of the hyphal wall protein 1 (*hwp1*) gene [24]. Moreover, to differentiate yeast isolates within *the C. haemulonii* complex, the *gpi* gene was amplified to detect the presence of *C. auris*. In addition, PCR products of approximately 400 bp were sequenced.

The species identified within the *C. parapsilosis* and *C. glabrata* complexes were differentiated using the method described by Arastehfar et al. (2018), which relies on size polymorphisms of the vacuolar membrane ATPase gene (V-ATPase) and ribosomal intergenic spacer (IGS) region, respectively [25]. To confirm the accurate identification of cryptic species and given the absence of controls for *C. nivariensis* and *C. bracarensis* within the *C. glabrata* complex, all ITS region amplification products from isolates identified as *C. glabrata* were sequenced on both strands using the same primer pairs employed in the amplification. The resulting PCR products were purified and sequenced by Psomagen^®^ (Rockville, MD, USA). The raw sequences were edited and assembled using Geneious^®^ Prime software v.2023.2.1 (Dotmatics, Boston, MA, USA). The sequences were submitted to GenBank, and accession numbers were assigned (https://www.ncbi.nlm.nih.gov). Accession numbers for the ITS rDNA region are provided in Table S2 of Supplementary Material S1. The primers and amplification conditions are provided in Table 1.

### 2.3. Hydrolytic Enzyme Activity Assays

The phospholipase and hemolytic activity of *Candida* isolates were evaluated following the methodology described by Neji et al. (2017) [28]. Similarly, proteolytic activity was assessed by measuring the enzymatic activity of caseinase, gelatinase, and the ability to hydrolyze bovine serum albumin (BSA), according to the procedures established in the same study [28]. Enzymatic activity was interpreted using the enzymatic activity coefficient (Pz), which is obtained by dividing the colony diameter (A) by the colony diameter plus the hydrolysis/precipitation zone (B) (Pz = A/B), as described in Price et al. (1982) [29]. The isolates were classified according to the Pz value into four categories: Pz = 1.0, no enzymatic activity; Pz = 0.99 to 0.90, weak enzymatic activity; Pz = 0.89 to 0.70, moderate activity; Pz ≤ 0.69, strong activity. The Pz values were averaged from two separate experiments, each performed in duplicate.

### 2.4. Biofilm Formation

Biofilm formation was carried out following the Saiprom et al. (2023) protocol [20], with minor modifications. Briefly, the isolates were cultured overnight in YPD at 37 °C, and a yeast suspension was prepared to a density equivalent to 0.5 McFarland standard. Moreover, a 100 μL aliquot of the suspension was added to each well of a 96-well flat-bottom plate and incubated at 30 °C for 48 h. After incubation, all wells were washed three times with PBS to remove non-adherent cells. The empty wells were allowed to dry for 45 min, and then 200 μL of 0.1% crystal violet was added to each well and incubated for 45 min at room temperature. After removing the crystal violet, the plate was air-dried at room temperature for 10 min. The wells were then gently washed twice with 200 μL of sterile distilled water. A volume of 500 μL of absolute ethanol was added to decolorize the biofilm, and the plate was incubated for 45 min at room temperature. A volume of 150 μL of eluted crystal violet was then transferred to a new 96-well plate, and the optical density (OD) at 590 nm was measured using a spectrophotometer (Thermo Scientific^TM^ Genesys 20, Oslo, Norway). Sterile YPD (yeast-free) was used as a negative control. Two independent experiments were conducted for each isolate, with each experiment being repeated three times. The biomass of each isolate was calculated as the mean optical density (OD) value derived from the two independent experiments. Biofilm production capacity was determined by averaging the ODs of each sample. The OD of the negative control (ODnc) was also measured. Strains were classified into four categories based on their OD values: non-producers (ODs ≤ ODnc), weak producers (ODnc < ODs ≤ 2 × ODnc), moderate producers (2 × ODnc < ODs ≤ 4 × ODnc), and strong producers (ODs > 4 × ODnc). *Candida albicans* ATCC 10231 was used as a positive control for biofilm production.

### 2.5. Statistical Analyses

Statistical analyses were performed using the R software v.4.3.1 (R Core Team, 2023) [30]. Figures were created using ggplot2 v.3.4.4 [31]. To assess significant differences in enzymatic activity among Candida species, a Kruskal–Wallis test was computed, followed by a post hoc Dunn test, and *p*-values were adjusted using the Benjamini–Hochberg method. To determine similarities in enzymatic activities among *Candida* species, a hierarchical clustering heatmap based on the Euclidean distance was generated with ComplexHeatmap v.2.18.0 following the guidelines developed by Gu et al. 2016 [32]. In all cases, *p*-values smaller than 0.05 were considered statistically significant.

## 3. Results

**Figure 2:**
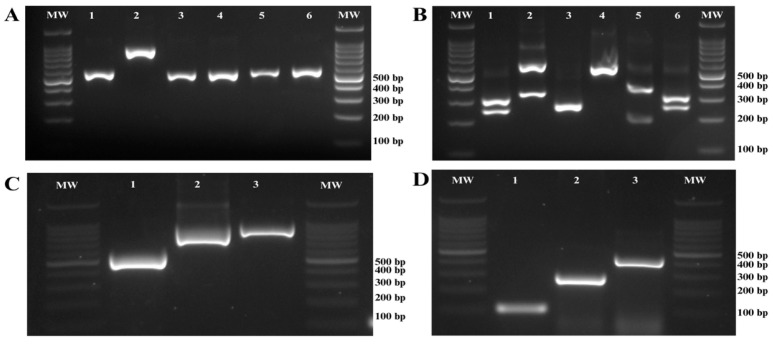
Agarose gel electrophoresis for the molecular identification of *Candida* species. (**A**) PCR amplification of the ITS region of ribosomal DNA. Line 1: *C. albicans*, Line 2: *C. glabrata*, Line 3: *C. krusei*, Line 4: *C. parapsilosis*, Line 5: *C. tropicalis*, Line 6: *C. dubliniensis*; (**B**) Digestion of ITS PCR products with the restriction enzyme MspI; Line 1: *C. albicans*, Line 2: *C. glabrata*, Line 3: *C. krusei*, Line 4: *C. parapsilosis*, Line 5: *C. tropicalis*, Line 6: *C. dubliniensis*. (**C**) PCR amplification targeting the *hwp1* gene for the identification of species within the *Candida albicans* complex; Line 1: *C. dubliniensis*, Line 2: *C. africana*, Line 3: *C. albicans*. (**D**) Molecular identification of species within the *Candida parapsilosis* complex using specific PCR. Line 1: *C. parapsilosis*, Line 2: *C. metapsilosis,* Line 3: *C. orthopsilosis.* Molecular weight marker (MW) size 100 bp.

A total of 80 non-duplicate *Candida* isolates were collected from four tertiary care hospitals in Honduras. Among the 80 isolates, 67 (83.7%) were successfully identified at both the genus and species levels, whereas 13 (16.2%) could only be identified at the genus level. Eleven isolates (13.8%) were misidentified when phenotypic results were compared with molecular data. Three isolates initially identified phenotypically as *C. albicans* were reidentified by molecular methods as *C. krusei* (*n* = 1) and *C. tropicalis* (*n* = 2). Two isolates originally classified as *C. haemulonii* were later correctly identified as *C. glabrata* and *C. parapsilosis*, respectively. Two isolates of *C. tropicalis* were incorrectly classified as *C. albicans*. In addition, two *C. krusei* isolates were misidentified as *C. parapsilosis* and *C. tropicalis*, respectively. Finally, misidentifications included one isolate originally classified as *C. albicans* and another as *C. zeylanoides*, both of which were later confirmed to be *C. parapsilosis*. Figure 2 presents the electrophoretic profiles of the genes employed to identify critical species and complexes within the *Candida* genus detected in this study.

Of the total isolates, 60 (75%) were obtained from San Pedro Sula and 20 (25%) from Tegucigalpa. In San Pedro Sula, 43 isolates (53.7%) were collected at Mario Catarino Rivas Hospital (HMCR-SPS), while 17 isolates (21.2%) were obtained from the Honduran Social Security Institute (IHSS-SPS). In Tegucigalpa, 14 isolates (17.5%) were collected from the Honduran Social Security Institute (IHSS-TGU), and 6 isolates (7.5%) were obtained from the National Cardiopulmonary Institute (INCP-TGU). When the sample collection sites were evaluated according to their geographical distribution, regional differences were observed. Most of the isolates were recovered from institutions in San Pedro Sula (HMCR-SPS and IHSS-SPS), where *C. tropicalis* and *C. parapsilosis* were the most frequently identified species, followed by *C. albicans*. Notably, *C. dubliniensis* was detected exclusively in this region. In contrast, in Tegucigalpa (IHSS-TGU and INCP), *C. albicans* and *C. tropicalis* were the predominant species. Although *C. glabrata* and *C. krusei* were identified in both regions, their overall prevalence was low.

**Figure 3:**
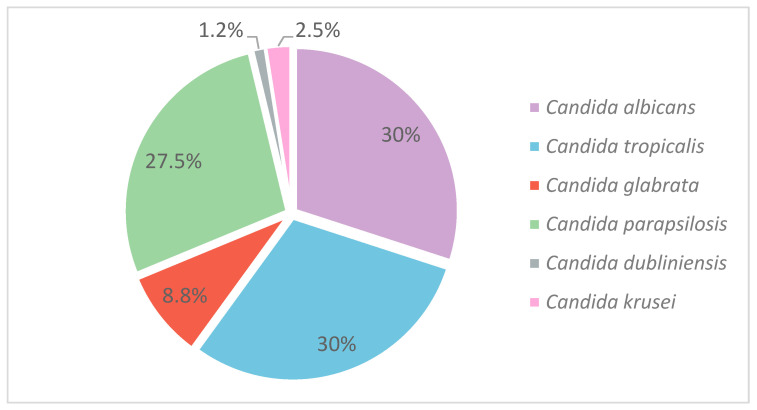
Distribution of *Candida* species.

The distribution of *Candida* species is shown in Figure 3. Supplementary Material S1, Figure S1 includes a map indicating the locations of the healthcare centers involved in this study.

Of the 80 isolates analyzed, 25 (31.2%) belonged to the *Candida albicans* complex. Within this group, 24 isolates (30%) were identified as *C. albicans* sensu stricto, and one isolate (1.2%) as *C. dubliniensis*. All isolates within the *C. parapsilosis* and *C. glabrata* complexes were classified as *C. parapsilosis* sensu stricto and *C. glabrata* sensu stricto, respectively. *C. albicans* and *C. tropicalis* were the most frequently detected species, each accounting for 24 isolates (30%). Non-albicans *Candida* species represented the remaining 70% of isolates, with *C. parapsilosis* (27.5%) and *C. glabrata* (8.7%) being the most prevalent following *C. tropicalis*. Less frequently detected species included *C. krusei* (2.5%) and *C. dubliniensis* (1.2%). Table S1, included in Supplementary Material S1, provides details on the distribution of isolates across healthcare centers.

**Figure 4:**
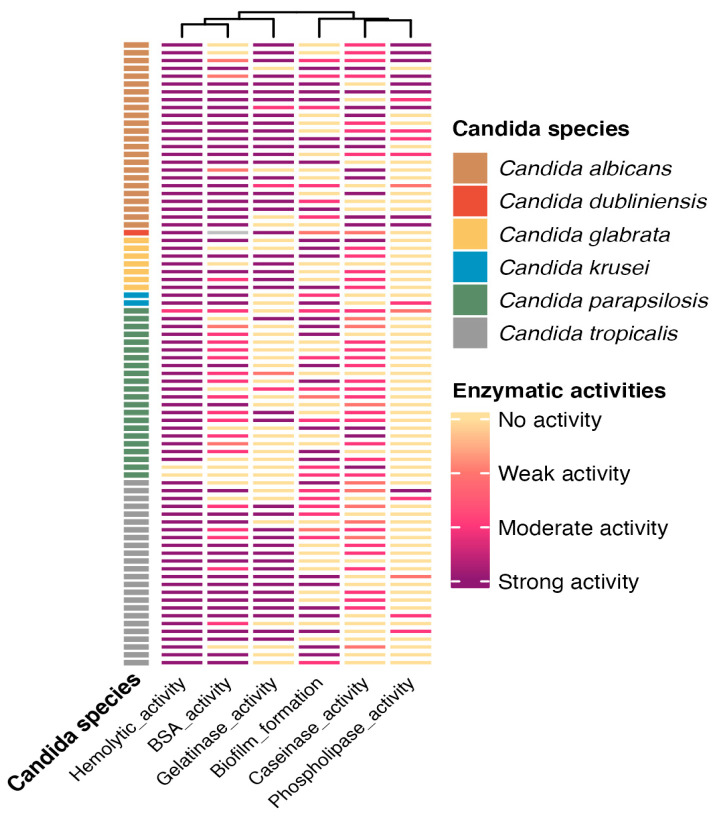
Heatmap showing the enzymatic activities of clinical *Candida* spp. isolates. The hemolytic activity profile, protease activity (BSA, gelatinase, and caseinase), biofilm formation, and phospholipase activity are shown for each isolate (rows), hierarchically clustered based on the similarity of their enzymatic activity patterns. Isolates are classified by species: *C. albicans* (brown), *C. dubliniensis* (orange), *C. glabrata* (yellow), *C. krusei* (blue), *C. parapsilosis* (green), and *C. tropicalis* (gray). Color intensity represents the enzymatic activity level: strong (dark purple), moderate (red), weak (light pink), or absent (light yellow).

**Figure 5:**
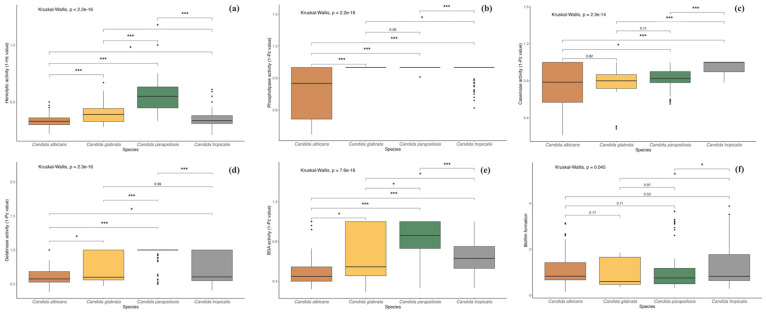
Boxplots illustrate the enzymatic activity levels of *Candida* species across different virulence groups. (**a**) Hemolytic activity; (**b**) Phospholipase activity; (**c**) Caseinase activity; (**d**) Gelatinase activity; (**e**) BSA activity; (**f**) Biofilm formation. The hard horizontal line within the boxplot indicates the median, the ends of the vertical lines represent the maximum and minimum values, and the black dots depict outliers. Asterisks denote statistically significant differences as determined by the post hoc Dunn test (* *p* < 0.05, *** *p* < 0.001).

Among the 80 isolates evaluated for hemolytic activity, 77 (96.3%) exhibited strong hemolysis. One isolate (1.2%) showed moderate activity, and two isolates (2.5%) demonstrated no hemolytic activity. Moreover, all isolates identified as *C. albicans*, *C. glabrata*, and *C. tropicalis* demonstrated 100% strong activity. Among the isolates identified as *C. parapsilosis*, 19 (86.3%) showed strong activity, 1 (4.5%) exhibited moderate activity, and 2 (9%) were non-hemolytic. Figure 4 provides a detailed overview of the virulence profiles obtained for each of the analyzed strains, grouped by species. Furthermore, Kruskal–Wallis test showed significant differences for all enzymatic activities among *Candida* species (*p* = 0.001). However, multiple comparisons revealed that hemolytic activity did not show variation between *C. albicans* and *C. tropicalis* (*p* > 0.05), and opposite patterns were observed for the other species (Figure 5a).

Among the 70 isolates tested for phospholipase activity, 10 (12.5%) exhibited strong activity, 16 (20%) moderate, 3 (3.7%) weak, and 51 (63.8%) showed no activity. *Candida albicans* exhibited the highest phospholipase activity among all species, with 14 isolates (17.5%) testing positive: 9 demonstrated strong activity, 4 moderate, and 1 weak. The second most active species, *C. tropicalis*, had five isolates (6.2%) with moderate activity. Additionally, one isolate of *C. tropicalis* and one of *C. parapsilosis* showed weak activity. Of the two *C. krusei* isolates, one showed moderate activity, while no *C. glabrata* isolates displayed phospholipase activity. Furthermore, post hoc testing revealed no difference between *C. glabrata* and *C. tropicalis*, whereas the remaining comparisons were statistically significant (*p* < 0.05; Figure 5b).

Proteolytic activity consisted in caseinase and gelatinase production, as well as BSA degradation. In the casein-containing medium, 57 isolates (71.2%) showed proteolytic activity, with *C. parapsilosis* (19 isolates, 23.7%), *C. albicans* (18 isolates, 22.5%), and *C. tropicalis* (13 isolates, 16.2%) exhibiting the highest activity. In the gelatinase test, 47 isolates (58.7%) showed activity, with *C. albicans* (20 isolates, 25%) and *C. tropicalis* (16 isolates, 20%) being the most active, while only 1 *C. parapsilosis* isolate showed weak activity. In the BSA medium, 67 isolates (83.7%) demonstrated activity, with *C. albicans* showing the highest activity (43 isolates), followed by *C. tropicalis* (16 isolates), and *C. parapsilosis* showing the lowest activity. For caseinase activity, multiple comparisons revealed no significant differences between *C. glabrata*, *C. albicans*, and *C. parapsilosis*, while gelatinase activity did not differ between *C. glabrata*, *C. albicans*, and *C. tropicalis* (*p* > 0.05). In contrast, all other comparisons were significant (*p* < 0.05), except between *C. glabrata* and *C. tropicalis* (Figure 5c–e).

Biofilm formation was detected in 79 isolates. Among these, 22 (27.5%) exhibited high activity, 32 (40%) showed moderate activity, and the remaining exhibited weak activity. *Candida tropicalis* demonstrated the highest biofilm production, followed by *C. albicans* and *C. parapsilosis*, with the latter showing the weakest values. Moreover, biofilm production statistically differed between *C. tropicalis* with *C. glabrata* and *C. parapsilosis* (*p* < 0.05; Figure 5f).

## 4. Discussion

In Honduras, an estimated 500,000 individuals have risk factors for severe fungal infections, and approximately 180,000 are believed to be affected by IFIs [33]. Specifically, the incidence of invasive *Candida* infections is estimated at 5 cases per 100,000 inhabitants, equating to roughly 495 cases per year [33,34]. However, this figure is likely underestimated due to limited access to diagnostic tools, such as mass spectrometry, molecular assays, and serological tests, as well as a shortage of adequately trained personnel, as previously reported [35].

According to the Joint United Nations Programme on HIV/AIDS (UNAIDS) estimates, approximately 40 million people are living with HIV worldwide [36]. In this regard, data from the Honduran Ministry of Health (SESAL) indicate that around 40,000 people living with HIV/AIDS in Honduras account for approximately 0.1% of the global burden [36,37]. Although this figure may seem relatively low on a global scale, it is highly significant in the national context, considering that the population of Honduras is approximately 10 million [38]. This translates to a national prevalence of around 0.4%, which is higher than the estimated global average, ranging between 0.2% and 0.3%. This situation is particularly relevant in the country, as HIV-associated immunosuppression is a major predisposing factor for the development of candidemia [39].

Diabetes mellitus is a well-established and increasingly important risk factor for invasive candidiasis [5,40]. Chronic hyperglycemia impairs innate immune responses, disrupts epithelial barriers, and promotes fungal overgrowth, thereby increasing host susceptibility to *Candida* infections [40]. Specifically, in the case of *C. albicans*, diabetes has been reported as the leading underlying condition in up to 70% of invasive candidiasis cases [40]. This is particularly concerning in Honduras, where, according to the Pan American Health Organization and the National Institute for Diabetes (INADI), more than one million people are estimated to have diabetes, nearly half of whom remain undiagnosed [41,42]. Notably, this high rate of undiagnosed diabetes coincides with the fact that approximately 18% of the Honduran population—equivalent to over 1.5 million individuals—has no access to any healthcare services, suggesting that a large proportion of diabetic patients may go unidentified and untreated [43,44].

Furthermore, around 4700 patients are currently undergoing hemodialysis, a procedure that inherently increases the risk of candidemia due to its invasive nature [45]. Collectively, these conditions create a highly conducive environment for the emergence and spread of *Candida* infections, emphasizing the urgent need to strengthen epidemiological surveillance, improve early diagnostic capabilities, and implement effective prevention strategies within the healthcare system. In this context, the present study aimed to evaluate the distribution of *Candida* species associated with candidemia in Honduras, to generate locally relevant data to support improved understanding and clinical management of this significant public health concern.

The epidemiology of *Candida* species varies significantly across geographic regions and can even differ between healthcare institutions within the same country [5,34,46]. In Honduras, the distribution of *Candida* species associated with IFIs has shown notable variability in recent years. In a 2013 multicenter study, Nucci et al. [47] investigated the epidemiology of candidemia in Latin America, including data from two hospitals in Tegucigalpa. That study reported a relatively low incidence of candidemia in Honduras compared to other countries in the region. *Candida albicans* was the most frequently isolated species, followed by *C. tropicalis*. Interestingly, *C. guilliermondii* emerged as the third most common species [47].

Later, Montes et al. (2019) analyzed 167 *Candida* isolates obtained between January and August 2018, of which 15 were associated with IFIs. *C. parapsilosis* and *C. albicans* were identified as the predominant species [23]. In response to the epidemiological shifts associated with the SARS-CoV-2 pandemic, a follow-up study was conducted in 2022, analyzing 38 isolates recovered from blood cultures between May 2021 and March 2022. In this study, *C. albicans* remained the primary etiological agent, followed by *C. parapsilosis*, *C. glabrata*, *C. krusei*, and *C. lusitaniae* [48].

Our findings confirm that *C. albicans* continues to be the leading cause of candidemia in Honduras, consistent with regional and global reports [6,16]. However, our data also reveal a shifting epidemiological pattern, with an increasing prevalence of non-albicans species, particularly *C. tropicalis*. This trend may be influenced by factors such as antifungal selective pressure, hospital-specific environmental conditions, or unique patient population characteristics. Further research is warranted to clarify the underlying risk factors contributing to species-specific candidemia [49,50,51].

Regarding the discrepancies observed in the identification of *Candida* species in this study, several studies have documented the limitations of currently available systems for yeast identification [52,53]. In the case of chromogenic media, the main limitation lies in the subjectivity of interpretation, as the accurate reading of color patterns largely depends on the experience of the technical personnel [23]. On the other hand, automated systems such as VITEK 2 and BD Phoenix have shown error rates of approximately 2% and 6%, respectively, in the identification of common yeasts [53,54]. These inaccuracies are partly due to the similarity of biochemical profiles among certain species, making them difficult to differentiate using systems based solely on phenotypic tests [55]. Moreover, both systems have reported error rates exceeding 25% when identifying uncommon yeasts [52,53,56]. These limitations have been attributed to shortcomings in the reference databases, particularly regarding new or emerging species such as *C. auris* and *C. vulturna* [56,57,58]. Technical factors also play a role, such as the quality of growth in culture, inoculum density, and its proper distribution within the automated system, all of which can negatively affect result accuracy [59]. In this context, the importance of continuous epidemiological surveillance is reinforced, along with the incorporation of complementary molecular methods that allow for precise species-level identification. This is crucial in the management of invasive fungal infections, where antifungal treatment must be tailored to the specific species involved due to differences in susceptibility and virulence. Furthermore, timely identification is especially relevant in the face of emerging and spreading species such as *C. auris*, which has had a significant global impact. In the case of Honduras, although its presence has not yet been reported within the national territory [22,48,60], adequate diagnostic capacity is essential for its eventual detection and containment.

**Figure 6:**
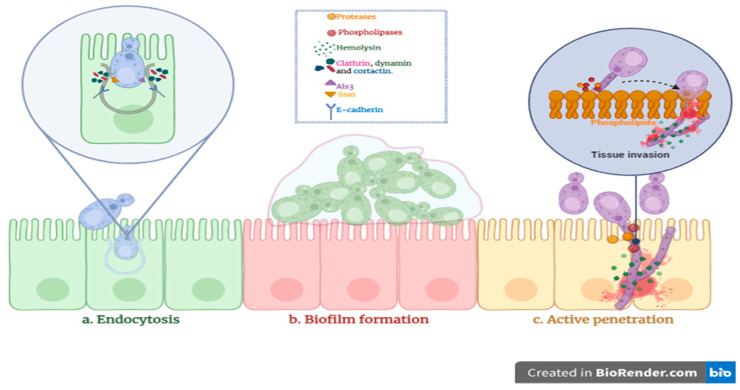
Three primary mechanisms contributing to *Candida* spp. invasion.

*Candida* species utilize several pathogenic mechanisms to invade host epithelial tissues (Figure 6). After initial adhesion, they can penetrate host cells through induced endocytosis or active penetration [61,62,63,64]. These processes are typically accompanied by the secretion of extracellular hydrolytic enzymes that degrade host tissues and facilitate invasion [18,63,64]. Additionally, biofilm formation enhances virulence by increasing resistance to antifungal agents and promoting persistent infections [18,65]. The present study evaluated multiple virulence factors in clinical *Candida* isolates, including hemolytic activity, phospholipase and protease production (caseinase, gelatinase, and BSA degradation), and biofilm-forming capacity.

Hemolysins are hydrolytic enzymes and key virulence factors in *Candida* species, enabling the lysis of erythrocytes and the release of iron, an essential micronutrient for fungal growth. This capability supports *Candida* proliferation in iron-restricted environments such as the bloodstream, promoting tissue invasion and contributing to host damage [66,67]. In our study, a high prevalence of strong hemolytic activity was observed among *Candida* isolates (96.3%), contrasting with findings from studies conducted in Brazil and Egypt, where only moderate activity was reported [21,68]. The study conducted in Egypt included isolates from various anatomical sites and was not limited to bloodstream infections. In contrast, the study from Brazil focused exclusively on isolates obtained from cases of candidemia. Conversely, our results are consistent with those of Tiwari et al. (2024) in northern India, where all the isolates were exclusively from cases of candidemia, demonstrated high hemolytic activity [69]. Similarly, Pandey et al. (2018) reported strong hemolytic activity in 95.8% of *C. albicans* and 100% of *C. tropicalis* isolates from candidemia cases in India [70]. Nouraei et al. (2020) also found strong hemolytic activity in *C. albicans* isolates associated with candidemia in Iran [71]. However, both the Indian and Iranian studies differ from our findings on NAC species isolated from blood, particularly *C. tropicalis*, as they reported a high degree of variability in hemolytic activity among species [70,71], whereas our study demonstrated uniformly strong activity among all identified species. Additionally, our results align with those reported by Neji et al. (2017) in Tunisia for *C. parapsilosis,* although that study included isolates from multiple clinical sources [28]. Similar concordance was noted with the findings of Saiprom et al. (2023) in Thailand, which involved candidemia isolates of *C. albicans*, *C. glabrata*, and *C. tropicalis* [20].

Phospholipases, another key group of hydrolytic enzymes, degrade lipid components of host cell membranes, promoting tissue invasion and damage [62,72]. In agreement with previous studies [20,62,71], all *C. albicans* isolates in our study exhibited strong phospholipase activity, reinforcing its role as one of the most virulent species in the *Candida* genus. Additionally, phospholipase activity was detected in *C. tropicalis* isolates, consistent with reports highlighting its capacity to secrete this enzyme and contribute to tissue damage [69,71]. However, in contrast to studies that reported a high frequency of phospholipase production in *C. parapsilosis* [20,71], only one isolate of this species showed such activity in our study. On the other hand, our findings corroborate those from northern India and Thailand, where no phospholipase activity was detected in *C. glabrata* isolates [20,69].

Proteases secreted by *Candida* species play a crucial role in pathogenesis by facilitating both tissue invasion and immune system evasion [28,63]. These hydrolytic enzymes can degrade structural and functional host proteins such as albumin, collagen, and mucin, thereby compromising mucosal and epithelial barriers and promoting surface colonization [20,66]. Simultaneously, they contribute to immune evasion by degrading key components of the host immune system, including antibodies, complement proteins, and cytokines [63,73]. This dual role—facilitating tissue destruction and modulating the immune response—makes proteases key virulence factors in the establishment and persistence of *Candida* infections [73]. In our study, *C. albicans* and *C. tropicalis* demonstrated notably higher proteolytic activity compared to other species. The elevated protease production in *C. albicans* is consistent with its well-established invasive potential, while the enzyme expression observed in *C. tropicalis* highlights its growing clinical relevance as a cause of invasive infections.

In addition, 98.7% of the isolates in our study demonstrated the ability to form biofilms, a major virulence factor that promotes the persistence of yeasts on medical devices, particularly central venous catheters, urinary catheters, and endotracheal tubes, and significantly contributes to antifungal resistance [65,74]. Biofilm formation complicates the eradication of infections and increases the risk of fungemia and other systemic complications [75]. Therefore, continuous monitoring is essential, particularly in patients with invasive devices, to effectively prevent and manage these infections.

Taken together, our preliminary analysis of species-specific virulence factors suggests low clonal diversity among *Candida* isolates associated with invasive fungal infections in Honduras. However, to confirm this hypothesis, additional studies incorporating molecular tools, such as microsatellite analysis or multilocus sequence typing (MLST), along with species-specific antifungal susceptibility profiling, are necessary. This combined approach has been effectively applied in studies conducted in Europe and Asia [76,77,78,79]. Implementing such methodologies would provide a more precise epidemiological characterization of *Candida* species and support the development of effective strategies for outbreak control and infection prevention across different hospital settings in the country.

## 5. Conclusions

*Candida albicans* remains the principal causative agent of candidemia in Honduras, followed by *C. tropicalis*. Our findings reflect a shifting epidemiological landscape, marked by an increasing prevalence of non-*albicans* species that exhibit diverse antifungal susceptibility and resistance profiles. These trends underscore the critical need to adopt accurate species identification methods and antifungal susceptibility testing as part of standard clinical practice. The results are consistent with global observations, confirming patterns such as the elevated expression of phospholipases and proteases in *C. albicans* and the notable biofilm-forming capacity of *C. tropicalis*. However, regional differences were also evident. For example, *C. parapsilosis* showed low phospholipase activity, emphasizing the importance of local surveillance to elucidate context-specific pathogenic traits. The observed interspecies variability in virulence factor expression reinforces the clinical relevance of species-level identification and phenotypic profiling. A comprehensive understanding of the pathogenic mechanisms employed by different *Candida* species is essential to refine diagnostic approaches, guide antifungal therapy, and inform infection control strategies. The predominance of highly virulent strains, particularly *C. tropicalis*, highlights the need for enhanced epidemiological monitoring and the implementation of robust infection prevention measures.

## Figures and Tables

**Table 1:** List of primers used in this study.

Primer Name	Locus	Sequence 5′ to 3′	Annealing Temperature °C	Reference
ITS1	ITS-ADNr	TCCGTAGGTGAACCTGCGG	56	[26]
ITS4		TCCTCCGCTTATTGATATGC		
CR-f	*hpw*1	GCTACCACTTCAGAATCATCATC	58	[24]
CR-r		GCACCTTCAGTCGTAGAGACG		
OM-f	VMA	GAGAAAGCACGCCTCTTTGC	60	[25]
OM-r		TCAGCATTTTGGGCTCTTGC		
P-f	ITS-ADNr	GCGGAAGGATCATTACAGAATG	60	
P-r		CTGGCAGGCCCCATATAG		
PCG-f	IGS	TCACTTTCAACTGCTTTCGC	60	
G-r		TGCGAGTCATGGGCGGAA		
N-r		ACCCCAGAGGCATAAATAGC	60	
B-r		GCAACTGGACGAAAGTGC		
03410_F	GPI	GCCGCTAGATTGATCACCGT	60	[27]
03410_R		TAGGTGTGGGTACCCTTGGT		

## Data Availability

The original contributions presented in this study are included in the article/Supplementary Material. Further inquiries can be directed to the corresponding author.

## Supplementary Materials

The following supporting information can be downloaded at: https://www.mdpi.com/article/10.3390/jof11070470/s1. Figure S1. Geographic distribution of the healthcare centers included in this study. The map indicates the locations of the participating institutions where *Candida* isolates were collected and analyzed. Table S1. Distribution of *Candida* species by healthcare institution. Table S2. Identification of *Candida glabrata* complex: GenBank accession numbers of sequenced isolates. Figure S2. Evaluation of the protease activity of *Candida* spp. on Sabouraud-based gelatin agar. The opaque areas around the colony show the presence of proteases capable of degrading gelatin. Figure S3. Evaluation of the hemolytic activity of *Candida* spp. on Sabouraud agar supplemented with human blood 6% and glucose 3%. The light areas reflect the yeast’s ability to lyse red blood cells. Figure S4. Evaluation of the protease activity of *Candida* spp. on Sabouraud agar supplemented with skim milk. The clearance zones indicate the activity of proteolytic enzymes on the medium. Figure S5. Evaluation of the protease activity of *Candida* spp. in Sabouraud-based BSA. Opaque areas around colonies indicate albumin degradation. Figure S6. Detection of phospholipase activity of *Candida* spp. in Sabouraud-based egg yolk agar. The opaque precipitate zones around the colony show enzymatic activity on the phospholipids of the egg yolk.
