# Supplementary material for: Virulence Factors and Molecular Identification of Candida Species Causing Candidemia in Honduras
Source: J Fungi (Basel). 2025 Jun 20;11(7):470. doi: 10.3390/jof11070470 (PMC12295617; doi:10.3390/jof11070470)
Supplement: Supplementary file 1 [file jof-11-00470-s001.zip › jof-3689548-supplementary.pdf]

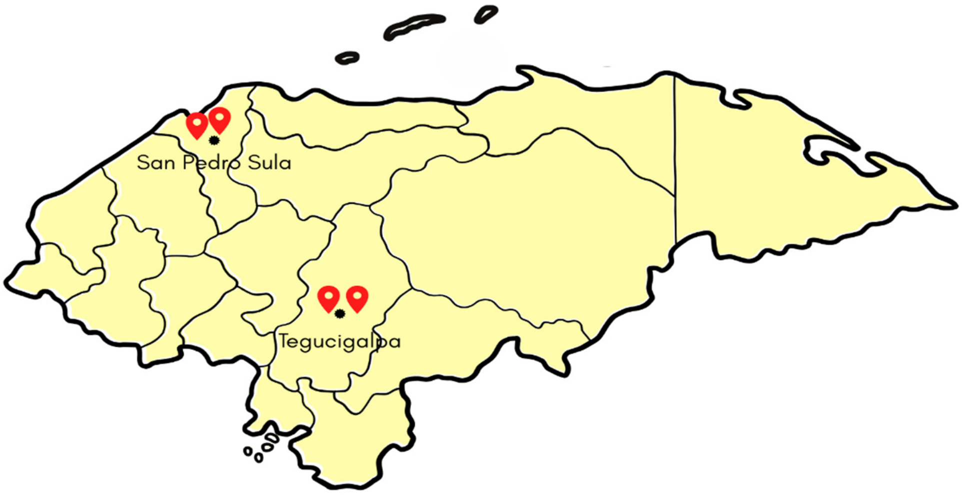

Figure S1. Geographic distribution of the healthcare centers included in this study. The map indicates the locations of the participating institutions where *Candida* isolates were collected and analyzed.

Table S1. Distribution of *Candida* species by healthcare institution

Institution	Identification method	n	*C. albicans*	*C. tropicalis*	*C. glabrata*	*C. parapsilosis*	*C. dubliniensis*	*C. krusei*
HMCR-SPS	VITEK®	43	13	16	1	11	1	1
IHSS-TGU	BD Phoenix™	14	5	4	3	1	0	1
IHSS-SPS	BD Phoenix™	17	4	1	2	8	0	0
INCP	Germ tuve/ CHROMagar™ Candida Plus	6	2	3	1	2	0	0
TOTAL		80	24	24	7	22	1	2

Table S2. Identification of *Candida glabrata* complex: GenBank accession numbers of sequenced isolates

Specie	**GenBank Accession Numbers Assigned**	**Query Cover**	**Per. Identity**	**Blast results (NCBI accession number)**
Candida glabrata	PP333224.1	100%	100%	*Nakaseomyces glabratus* (MN902081.1)
Candida glabrata	PP334144.1	100%	100%	*Nakaseomyces glabratus* (MN902081.1)
Candida glabrata	PP334148.1	100%	100%	*Nakaseomyces glabratus* (MK998697.1)
Candida glabrata	PP334180.1	100%	100%	*Nakaseomyces glabratus* (MT548914.1)
Candida glabrata	PP334431.1	100%	100%	*Nakaseomyces glabratus* (MH545922.1)
Candida glabrata	PP334486.1	100%	100%	*Nakaseomyces glabratus* (MN902081.1)
Candida glabrata	PV680018	100	100	*Nakaseomyces glabratus* (MF187288.1)

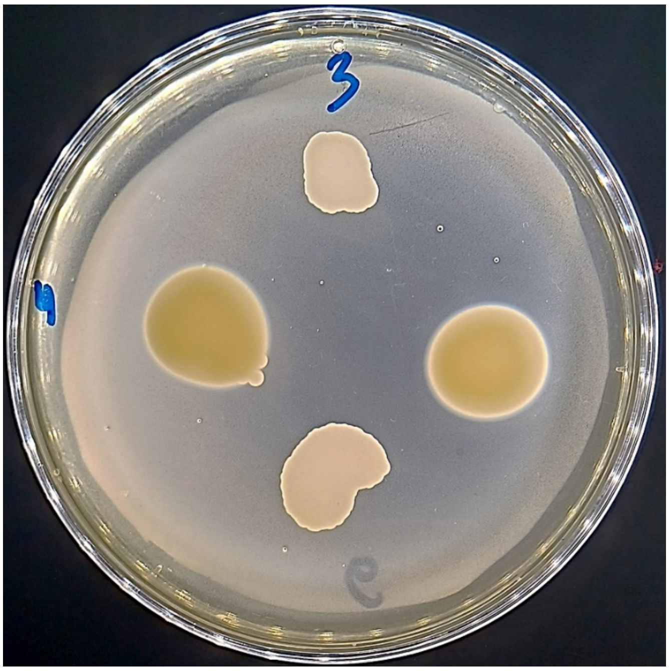

Figure S2. Evaluation of the protease activity of *Candida* spp. on Sabouraud-based gelatin agar. The opaque areas around the colony show the presence of proteases capable of degrading gelatin.

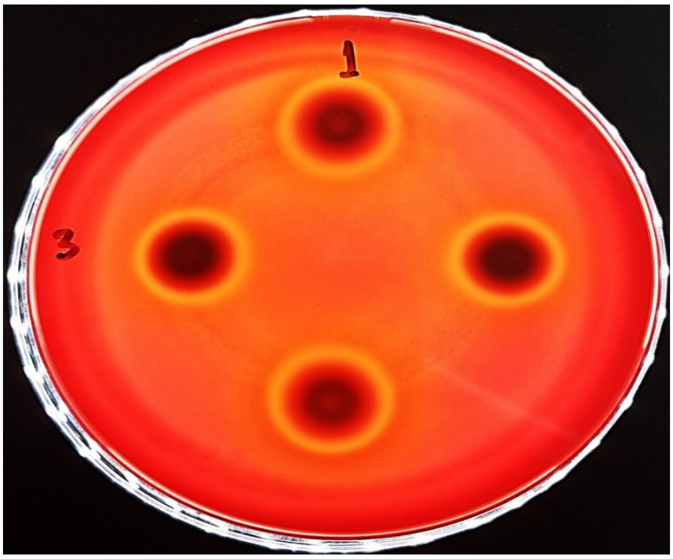

Figure S3. Evaluation of the hemolytic activity of *Candida* spp. on Sabouraud agar supplemented with human blood 6% and glucose 3%. The light areas reflect the yeast's ability to lyse red blood cells.

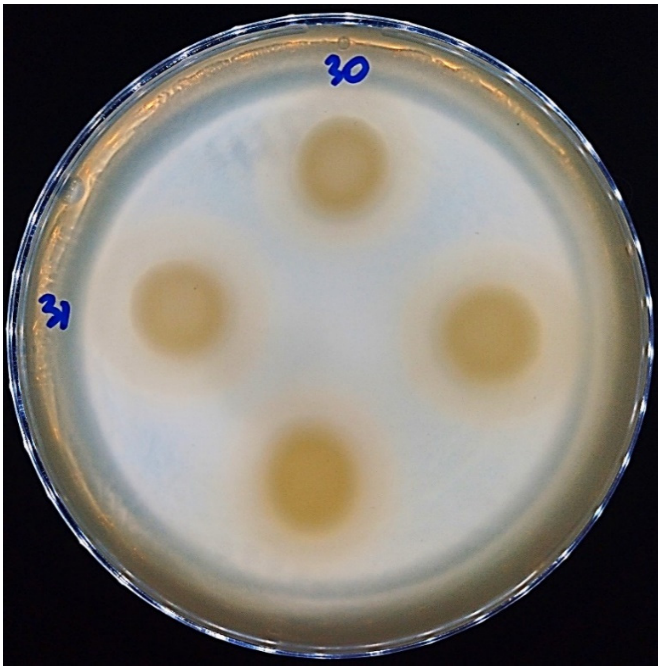

Figure S4. Evaluation of the protease activity of *Candida* spp. on Sabouraud agar supplemented with skim milk. The clearance zones indicate the activity of proteolytic enzymes on the medium.

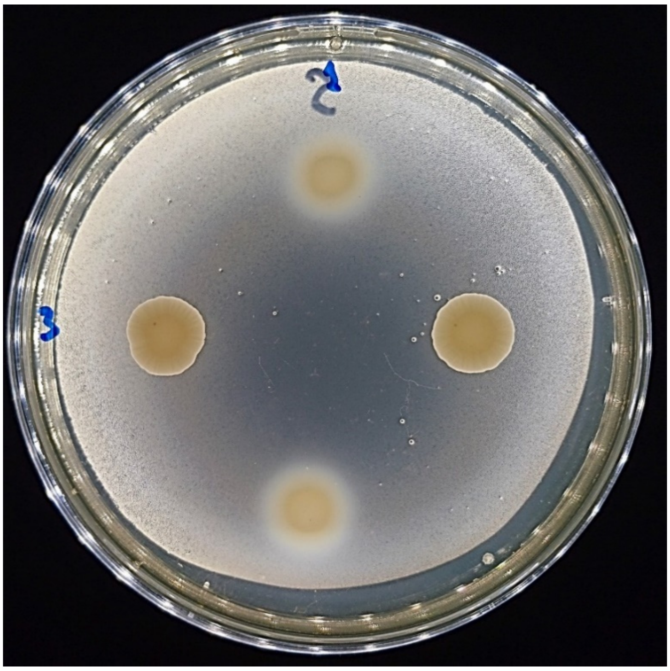

Figure S5. Evaluation of the protease activity of *Candida* spp. in Sabouraud-based BSA. Opaque areas around colonies indicate albumin degradation.

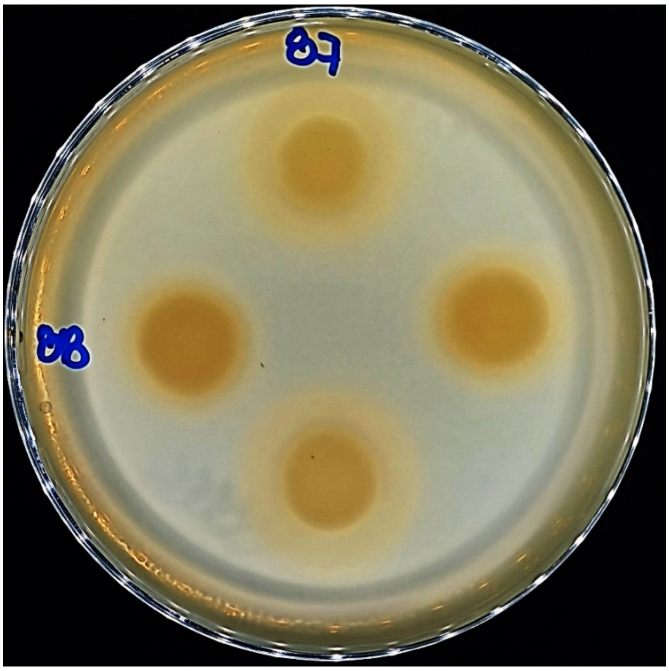

Figure S6. Detection of phospholipase activity of *Candida* spp. in Sabouraud-based egg yolk agar. The opaque precipitate zones around the colony show enzymatic activity on the phospholipids of the egg yolk.
